# Orchestrating cancer stem cell plasticity: deubiquitinases as key regulators of EMT and metabolic rewiring

**DOI:** 10.3389/fmed.2026.1957953

**Published:** 2026-10-16

**Authors:** Tao Qin, Yupeng Wang, Minhao Li, Qian Liu, Lu Yue, Shuzhen Liang, Duoping Sun

**Affiliations:** 1 The Third Clinical School of Medical Science, Qingdao University, Qingdao Municipal Hospital, Qingdao, China; 2 Department of Oncology, Qingdao Hospital, University of Health and Rehabilitation Sciences (Qingdao Municipal Hospital), Qingdao, China; 3 Pharmacy Department, Qingdao Eighth People’s Hospital, Qingdao, China; 4 Supervision Office (Ministry of Justice), Qingdao Central Hospital, University of Health and Rehabilitation Sciences (Qingdao Central Hospital), Qingdao, China; 5 Central Laboratories, Qingdao Municipal Hospital, University of Health and Rehabilitation Sciences, Qingdao, China; 6 Department of Radiation Oncology, Qingdao Hospital, University of Health and Rehabilitation Sciences (Qingdao Municipal Hospital), Qingdao, China

**Keywords:** cancer stem cells, deubiquitinases, epithelial-mesenchymal transition, metabolic reprogramming, therapeutic resistance, ubiquitin-proteasome system

## Abstract

Cancer stem cells (CSCs) drive tumor relapse, metastasis, and multidrug resistance, primarily attributed to their profound phenotypic plasticity. This plasticity largely depends on the dynamic interplay between the epithelial-mesenchymal transition (EMT) program and metabolic reprogramming. However, the molecular mechanisms coordinating these processes remain incompletely elucidated. Emerging evidence suggests that deubiquitinating enzymes (DUBs) may participate in regulating this complex crosstalk. This review systematically elaborates on the functional roles of DUBs in maintaining CSC characteristics within malignancies, exploring how they regulate CSC fate by modulating developmental signaling cascades (Wnt/β-catenin, Hedgehog (Hh), Notch, and TGF-β). By preventing the proteasomal degradation of key transcription factors, DUBs simultaneously drive EMT and coordinate metabolic reprogramming to meet the energetic demands of migrating CSCs. Furthermore, we emphasize the bidirectional feedback loops between EMT-TFs and DUBs, which cooperatively amplify stemness. Finally, selective targeting of the DUB-EMT-metabolism axis may represent a potential strategy for dismantling CSC plasticity and improving responses to anticancer therapies.

## 1 Introduction

Cancer stem cells (CSCs) are increasingly recognized as the primary drivers of tumor heterogeneity, as well as key forces behind recurrence, distant metastasis, and therapeutic resistance (1). Emerging evidence suggests that CSCs establish specialized niches within the tumor microenvironment, maintaining their stem-like properties through dynamic interactions with fibroblasts, immune cells, and the extracellular matrix. This unique microenvironment further enhances their resistance to therapy. Although conventional chemotherapy and radiotherapy can eliminate a large number of proliferating tumor cells, they often fail to completely eradicate CSCs, leading to recurrence, metastasis, and cancer-related mortality (2). A distinctive feature of CSCs is their remarkable plasticity. Beyond self-renewal and tumor initiation, CSCs can reversibly shift between different phenotypic states in response to microenvironmental cues and therapeutic stress (3). Among the processes underlying this plasticity, epithelial-mesenchymal transition (EMT) has received particular attention. Through EMT, epithelial tumor cells lose apical-basal polarity and intercellular adhesion, while upregulating mesenchymal markers such as Vimentin (VIM) and Snail. To support the energetic demands of metastasis, tumor cells undergo metabolic reprogramming and enhance their migratory capacity by activating pathways such as Wnt/β-catenin and TGF-β (4).

Clinical evidence indicates that tumors containing a high proportion of CSCs are significantly associated with poor prognosis. In breast cancer, CSC enrichment increases the 3-year recurrence risk by 2.8-fold and significantly shortens progression-free survival (PFS) (5). Similarly, a colorectal cancer cohort study linked elevated CD133⁺ CSC levels to a 42% decrease in 5-year survival rates. On this basis, putative CSC-associated markers such as ALDH1 and CD44 have been investigated for their potential prognostic value. These observations also provide a rationale for further evaluating the CSC regulatory network as a potential therapeutic target, although its clinical utility remains to be established.

Molecularly, EMT is characterized by the loss of epithelial adhesion molecules, particularly E-cadherin (CDH1), and the concomitant upregulation of mesenchymal markers like VIM and N-cadherin (CDH2). This process, which confers migratory and invasive properties on cells, is primarily driven by EMT transcription factors (EMT-TFs), including the Snail (Snail1/2), Twist (Twist1/2), and ZEB (ZEB1/2) families (6). Emerging evidence underscores a bidirectional regulatory network between EMT and metabolic reprogramming in CSCs, which is crucial for maintaining tumor stemness. EMT-TFs can directly drive metabolic shifts by modulating enzyme expression; for instance, Twist1 enhances glycolysis in lung cancer via the Wnt/β-catenin pathway (7). Furthermore, Twist1 is a key regulator of aerobic glycolysis in pancreatic ductal adenocarcinoma (PDAC); its depletion severely impairs glucose consumption, lactate secretion, and extracellular acidification (8). Conversely, the metabolic intermediates generated by these reprogramming events can retroactively regulate EMT. For instance, in hypoxic PDAC cells, hypoxia-inducible factor-1α (HIF-1α) upregulates glycogen phosphorylase L (PYGL) in the quasi-mesenchymal subpopulation. PYGL mobilizes glycogen reserves to fuel glycolysis, thereby promoting EMT progression (9). Similarly, aldehyde dehydrogenase 1A3 (ALDH1A3) sustains CSC stemness and coordinates EMT by balancing oxidative phosphorylation (OXPHOS) and glycolysis while maintaining reactive oxygen species (ROS) levels (10).

The ubiquitin-proteasome system (UPS) is essential for intracellular protein homeostasis. As a key component of the UPS, deubiquitinating enzymes (DUBs) reverse ubiquitination modifications by hydrolyzing ubiquitin chains on target proteins, thereby modulating their stability, subcellular localization, and activity (11). Consequently, DUB dysregulation causes aberrant accumulation of downstream substrates, including EMT-TFs and metabolic enzymes, ultimately driving tumor initiation and progression. To date, approximately 100 DUBs have been identified and classified into six structural families: ubiquitin-specific proteases (USPs), ubiquitin C-terminal hydrolases (UCHs), ovarian tumor proteases (OTUs), Machado-Joseph disease proteases (MJDs), JAB1/MPN/Mov34 metallo-proteases (JAMMs), and the MINDY family (12). Due to the large number of USP family members and their strong association with cancer, it remains the most extensively studied family. Notably, USP2, USP10, and USP37 are frequently overexpressed in CSCs of various malignancies, where they maintain stemness by stabilizing specific EMT-TFs or metabolic enzymes (13–15).

Although growing evidence indicates that DUBs independently modulate EMT and tumor metabolism, a comprehensive framework integrating their roles in the bidirectional crosstalk between these processes in CSCs is still lacking. Specifically, how DUBs may coordinate this interaction across different tumor types—and whether such regulation follows pan-cancer or context-dependent patterns—remains unclear. To examine this issue, this review summarizes the functional characteristics of DUBs and their molecular roles in EMT-metabolism crosstalk. Furthermore, we propose a multilayered regulatory model in which DUBs may act as regulatory nodes connecting EMT activation, metabolic rewiring, epigenetic regulation, and ferroptosis evasion, thereby potentially contributing to multidrug resistance and tumor progression (Figure 1). Rather than conceptualizing DUBs as static enzymes interacting with single substrates, we emphasize that their biological effects are dictated by dynamic substrate selection across distinct cellular states. DUB substrate specificity is determined by multiple factors, including subcellular localization, cell-type-specific expression patterns, adaptor and scaffold proteins, ubiquitin-chain linkage preferences, post-translational modifications, and the signaling landscape of the tumor microenvironment. These variables explain both why the same DUB can target different substrates in distinct microenvironments and why individual DUBs can exert either oncogenic or tumor-suppressive functions in a context-dependent manner. For instance, this explains how USP7 (also known as HAUSP) differentially regulates β-catenin, DNA methyltransferase 1 (DNMT1), glioma-associated oncogene homolog (Gli), or transformation/transcription domain-associated protein (TRRAP) depending on the specific molecular context. Consequently, this review transcends a systematic catalog of DUB-substrate pairs to provide a framework for understanding how DUB-dependent regulatory networks modulate CSC plasticity, metastatic progression, and therapeutic sensitivity.

**Figure 1:**
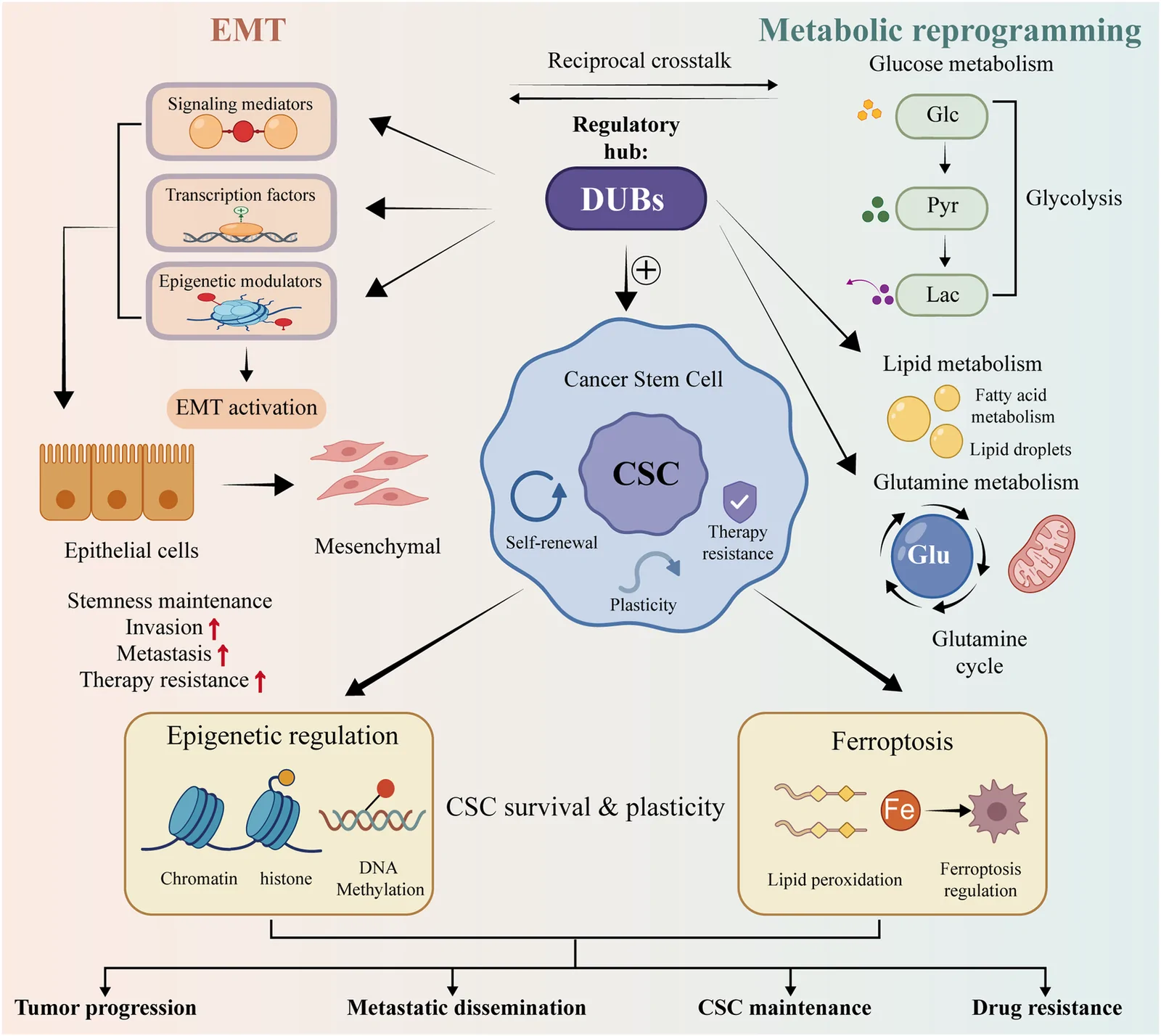
DUBs serve as a central molecular hub orchestrating EMT, metabolic reprogramming, and stemness in cancer. By dynamically preventing the ubiquitin-proteasomal degradation of critical substrates, DUBs function as central regulators of the reciprocal crosstalk between the EMT program and metabolic rewiring in CSCs. DUBs stabilize EMT-inducing transcription factors, signaling mediators, and epigenetic modulators, thereby driving EMT activation, sustaining stemness, and promoting invasion and metastasis. Concurrently, DUBs directly modulate key metabolic enzymes involved in glycolysis, lipid metabolism, and glutaminolysis, equipping CSCs with the metabolic plasticity required to survive energetic stress. Furthermore, DUB-mediated epigenetic remodeling and regulation of lipid peroxidation govern ferroptosis sensitivity, conferring profound resistance to conventional therapies. Ultimately, this intricate, multilayered DUB–EMT–metabolism regulatory network actively drives tumor progression, metastatic dissemination, and multidrug resistance.

## 2 Expression and functions of DUBs in CSCs

Glioblastoma (GBM) is the most aggressive astrocytic neoplasm of the central nervous system (CNS). Within GBM, glioblastoma stem cells (GSCs) represent a functionally distinct subpopulation with tumor-initiating capacity. These cells are the major contributors of tumor recurrence, therapeutic refractoriness, and metastasis, making them important therapeutic targets (16).

Ubiquitin-Specific Protease 1 (USP1) is aberrantly overexpressed in primary human GBM tissues and patient-derived GSCs (16). Mechanistically, USP1 maintains the GSC phenotype by stabilizing two key substrates: Inhibitor of Differentiation 1 (ID1) and Checkpoint Kinase 1 (CHEK1). ID1 promotes stemness by upregulating the pluripotency factor SOX2 and downregulating the kinase inhibitor p21^Cip1/Waf1^ (17). Meanwhile, CHEK1 is crucial for the DNA damage response (DDR) cascade; its activated form (p-CHEK1) is maintained at high intracellular levels by USP1, thereby driving GSC radioresistance (18). USP1 overexpression induces a significant shift toward a stem-like state, characterized by elevated expression of SOX2 and Olig2, alongside suppression of the astrocytic differentiation marker GFAP and the cell cycle regulator p21. Notably, ID1 and p-CHEK1 are significantly enriched in GSCs compared to the bulk tumor population (16). Collectively, USP1 maintains GSCs and drives radiotherapy resistance by stabilizing regulators of both stemness and DDR signaling pathways, highlighting its translational potential as a novel therapeutic target for GBM.

Breast cancer stem cells (BCSCs) drive tumor initiation, metastatic dissemination, therapeutic resistance, and relapse. Emerging evidence indicates that DUBs maintain BCSCs by modulating epigenetic regulators and stemness-associated signaling networks. For instance, lysine-specific demethylase 1 (LSD1) is an epigenetic regulator controlling H3K4 methylation dynamics, and it is enriched at genomic loci governing pluripotency and differentiation blockade. USP28 directly stabilizes LSD1 via deubiquitination, thereby enhancing the stem-like features of breast cancer cells (19). Accordingly, depletion of USP28 upregulates differentiation-associated genes, including *p21^Cip1/Waf1^, HNF4*, *HoxA10*, and *FoxA2*. Thus, the USP28-LSD1 axis maintains the undifferentiated state and self-renewal capacity of BCSCs by antagonizing the differentiation program (19).

USP44 is closely associated with an aggressive BCSC phenotype. Specifically, the USP44-positive BCSC subpopulation promotes vasculogenic mimicry, a phenomenon tightly linked to tumor invasiveness and poor prognosis (20). Clinically, enrichment of USP44^+^ tumor stem-like cells is associated with poor patient outcomes, indicating that USP44 serves as both a functional driver of BCSC aggressiveness and a prognostic biomarker (21).

Beyond GBM and breast cancer, DUBs also play key roles in regulating CSC phenotypes in various malignancies. In renal cell carcinoma, for example, USP21 is significantly upregulated in stem-like cells, driving self-renewal through two complementary mechanisms. First, it binds the interleukin-8 (IL-8) promoter to directly upregulate its transcription (22). Second, it stabilizes Gli1 via a protein kinase A (PKA)-dependent phosphorylation cascade, thereby activating the Hedgehog (Hh) signaling pathway. Similarly, in colorectal cancer, USP54 is significantly enriched in CSCs and is associated with shortened overall survival, although the specific mechanism by which it mediates CSC maintenance remains to be elucidated (23). Collectively, these findings establish the concept that DUBs orchestrate stem-like phenotypes by regulating transcriptional programs, DDR signaling, developmental pathways, and epigenetic states, further confirming their potential as viable therapeutic targets.

## 3 DUB-mediated crosstalk between EMT and CSCs

### 3.1 Functional intersection of EMT and CSCs

Substantial evidence indicates a profound interconnection between EMT and CSC characteristics: cells undergoing EMT frequently acquire stem-like properties, while CSCs reciprocally exhibit EMT markers along with enhanced migratory and invasive capabilities (24). In mammary epithelial cells (MECs), induction of EMT generates a transcriptome resembling that of mesenchymal stem cells, conferring multilineage differentiation and augmented wound-healing potential. Consistent with this, Mani et al. demonstrated that inducing EMT in MECs expands the CD44^+^/CD24^−^ stem-like population and endows it with tumor-initiating capacity (25). Crucially, these phenotypic transitions are accompanied by extensive metabolic rewiring—highlighting the tight integration of EMT, stemness, and metabolism. Similarly, in prostate cancer, cancer-associated fibroblasts not only induce EMT in tumor cells but also enhance stemness markers, sphere-forming ability, and self-renewal capacity (26, 27). In summary, these findings underscore the biological interdependence and coordinated regulation between EMT and CSC programs.

### 3.2 Key DUBs in the regulation of EMT

DUBs may participate in the EMT-CSC crosstalk by modulating the stability of several signaling intermediates, transcription factors, and chromatin regulators. For example, the tumor-suppressive CYLD attenuates NF-*κ*B signaling. In esophageal cancer stem-like cells, the STAT3/miR-181b axis downregulates CYLD, thereby enhancing proliferation and anti-apoptotic capacity (28). Simultaneously, loss of CYLD potentiates Notch signaling (29), which upregulates Snail and N-cadherin to promote the EMT process. Similarly, USP7, as a multifaceted DUB, plays a central role in both CSC maintenance and EMT. It maintains stemness by deubiquitinating TRRAP, thereby promoting the expression of stemness-related genes such as *MYC* (30). In epithelial ovarian cancer, USP7 acts synergistically with the E3 ligase MARCH7 to suppress E-cadherin and upregulate β-actin, driving canonical EMT events (31). Furthermore, USP7 interacts with the EMT-inducing transcription factor ZEB1; under genotoxic stress, ZEB1 facilitates USP7-mediated CHK1 stabilization, enhancing homologous recombination repair and conferring radioresistance. Overall, USP7 serves as a regulatory node that connects stemness self-renewal, EMT, and stress tolerance across different tumor backgrounds through substrate-specific mechanisms.

In addition to serving as a potential CSC marker in various cancers, ubiquitin-specific protease 22 (USP22) plays a critical role in coordinating transcriptional networks, driving Myc-induced transformation, and regulating cell-cycle progression. It also promotes the maintenance of CSC properties by sustaining Hes1 expression (32). Its depletion induces G1-phase arrest, highlighting its significant role in proliferation. In pancreatic cancer, USP22 drives EMT by upregulating N-cadherin, VIM, ZEB1, and Snail, while simultaneously suppressing E-cadherin (33). Mechanistically, USP22 deubiquitinates and stabilizes SIRT1, which subsequently inhibits STAT3 acetylation and modulates downstream targets such as MMP9 and Twist. In colon cancer cells, USP22 silencing similarly disrupts this SIRT1/Twist/MMP9 axis (34), thereby reinforcing its role in coupling stemness, EMT, and invasive behavior.

Overall, aberrant DUB activity not only enhances CSC properties but also strengthens functional crosstalk between CSCs and EMT by stabilizing EMT regulators. Consequently, selectively targeting specific DUBs has emerged as a potential therapeutic strategy aimed at dissociating these phenotypes and inhibiting tumor metastasis and relapse. Notably, functional redundancy among DUBs is a critical confounding factor in both mechanistic exploration and translational research. For instance, in the β-catenin signaling cascade, multiple DUBs have been reported to stabilize the same pathway effector molecules across diverse tumor backgrounds. This convergence suggests that the phenotypic impact of inhibiting a single DUB may vary significantly depending on tumor type, CSC status, and underlying signaling network architecture. In certain scenarios, a specific DUB may serve as the dominant enzyme for β-catenin deubiquitination due to differential expression levels, subcellular localization, or preferential recruitment of specific scaffold proteins; in such instances, single-agent inhibition alone may be sufficient to disrupt EMT and stemness. Conversely, in other contexts, parallel DUB activities may buffer pathway output, where blockade of one enzyme is partially compensated by others, resulting in limited therapeutic efficacy. Therefore, the effectiveness of DUB-targeted therapies depends not merely on whether a given DUB can deubiquitinate β-catenin, but more critically on whether that DUB acts as a non-redundant signaling node within a specific cellular milieu. To circumvent CSC plasticity, future DUB-targeted therapies should integrate biomarker-driven stratification strategies to precisely identify rate-limiting DUBs, combined with combinatorial approaches to overcome overlapping compensatory networks.

### 3.3 Distribution of EMT-related DUB targets in different tumors

More than 90% of human cancers originate from epithelial cells, which form tightly packed cell layers through specialized junctional structures such as tight junctions and adherens junctions. In contrast, mesenchymal cells lack organized layering, exhibit loose contact with neighboring cells, and possess strong migratory capacity. EMT refers to the process by which epithelial cells transform into mesenchymal cells, representing a hallmark of tumor invasion and metastasis (35). Phenotypically, EMT is characterized by the dissolution of intercellular junctions and increased motility; molecularly, it is defined by the suppression of epithelial proteins like E-cadherin and the induction of mesenchymal markers such as VIM (36). DUBs are extensively involved in the EMT process across various tumor types. Table 1 summarizes the currently reported EMT-associated DUBs and their major targets in different cancers.

**Table 1:** EMT-associated DUBs and their representative target proteins in different cancer types.

EMT-associated DUBs	Cancer types	Representative target protein(s)	EMT regulatory direction	Ubiquitin chain specificity	Level of experimental validation	Reference(s)
USP1	Ovarian Cancer	Snail	Promotes EMT/stemness	NA	Cellular	(86)
USP2	TNBC	Twist1	Promotes EMT	K48-linked polyubiquitination	Cellular/animal	(13)
USP3	GBM	Snail	Promotes EMT	NA	Cellular/animal	(145)
USP4	CRC; HCC	PRL-3; β-catenin; TGF-β; Twist1	Promotes EMT	NA	Cellular/animal	(65, 74, 146, 147)
USP5	CRC; Bladder Cancer	Snail; Twist1	Promotes EMT	NA	Cellular/animal	(148, 149)
USP7	Ovarian; Bladder Cancer； ESCC	MARCH7; HIF-1α; β-catenin;	Promotes EMT	K48-linked polyubiquitination	Cellular/animal	(31, 135, 150)
USP8	Breast Cancer	TβRII	Promotes EMT	NA	Cellular/animal	(151)
USP9X	TNBC	Snail	Promotes EMT	K48-linked polyubiquitination	Cellular/animal	(152)
USP10	Osteosarcoma; CRC；	YAP1; ZEB1;	Promotes/ Inhibits EMT	Removes K27-linked polyubiquitination	Cellular/animal	(153, 154)
USP11	Breast cancer	TβRII; TβRI	Promotes EMT	K48-linked polyubiquitination	Cellular/animal	(155–157)
USP13	Gastric Cancer	Snail	Promotes EMT	NA	Cellular	(158)
USP14	Gastric cancer	Vimentin	Promotes EMT	NA	Cellular	(159)
USP15	Glioblastoma	TβR-I	Promotes EMT	NA	Cellular/animal	(67)
USP18	ESCC	ZEB1	Promotes EMT	NA	Cellular/animal	(160)
USP20	HNSCC	CTSL	Promotes EMT	K48-linked polyubiquitination	Cellular/animal	(161)
USP21	Laryngeal Cancer	YY1	Promotes EMT	NA	Cellular	(162)
USP22	iCCA; TNBC; NSCLC	SIRT1; c-Myc	Promotes EMT	NA	Cellular	(163–165)
USP27X	Breast Cancer; Pancreatic cancer	Snail	Promotes EMT	NA	Cellular	(166)
USP28	Breast Cancer	Snail	Promotes EMT	NA	Cellular/animal	(167)
USP29	Gastric Cancer; TNBC; Lung Adenocarcinoma	Snail; Twist1	Promotes EMT	K48-linked polyubiquitination	Cellular/animal	(73, 168, 169)
USP32	CRC	SEMA4C	Promotes EMT	K48-linked polyubiquitination	Cellular	(170)
USP33	TNBC	TAP63	Inhibits EMT	K48-linked polyubiquitination	Cellular/animal	(171)
USP35	Gastric Cancer	Snail	Promotes EMT	Removes K6/K11/K27/K33/K48/K63-linked polyubiquitin chains	Cellular/animal	(172)
USP37	Breast Cancer; Lung Cancer	Gli1; Snail	Promotes EMT	NA	Cellular/animal	(15, 173)
USP38	Melanoma	β-catenin	Promotes EMT	K48-linked polyubiquitination	Cellular/animal	(174)
USP39	HCC	ZEB1	Promotes EMT	K48-linked polyubiquitination	Cellular/animal	(175)
USP44	Colorectal cancer	Axin1	Inhibits EMT	NA	Cellular/animal	(176)
USP47	CRC	Snail	Promotes EMT	NA	Cellular/animal	(177)
USP48	NSCLC	β-catenin	Promotes EMT	NA	Cellular/animal	(178)
USP51	Gastric Cancer; Breast Cancer	ZEB1	Promotes EMT	NA	Cellular/animal	(179, 180)
UCH-L1	NSCLC	Twist1	Promotes EMT	Removes K11/K63-linked poly-Ub chains	Cellular/animal	(85)
UCH-L3	Ovarian cancer	Snail	Promotes EMT	NA	Cellular	(181)
OTUB1	ESCC; CRC; Glioma; Breast Cancer; Ovarian Cancer; Gastric Adenocarcinoma	ZEB1; β-catenin; N-cadherin; Vimentin; E-cadherin; Smad2/3; Snail; FOXM1	Promotes EMT	NA	Cellular/animal	(182–184)
OTUB2	iCCA	β-catenin	Promotes EMT	NA	Cellular/animal	(185)
A20(TNFAIP3)	Breast Cancer	Snail	Promotes EMT	NA	Cellular/animal	(186)
DUB3	Breast Cancer	Slug (Snail2), Twist1	Promotes EMT	NA	Cellular/animal	(187)
BRCC3	TNBC	ZEB1	Promotes EMT	NA	Cellular/animal	(188)
CYLD	ESCC; T-ALL; BCC; Breast cancer	STAT3; NF-*κ*B; Snail, Gli1; TGF-β/SMAD; YAP1	Inhibits EMT	NA	Cellular/animal	(28, 55, 189, 190)

CSCs, cancer stem cells; EMT, epithelial-mesenchymal transition; EMT-TFs, EMT transcription factors; DUBs, deubiquitinating enzymes; UPS, ubiquitin-proteasome system; DDR, DNA damage response; ROS, reactive oxygen species; USPs, ubiquitin-specific proteases; UCHs, ubiquitin C-terminal hydrolases; OTUs, ovarian tumor proteases; MJDs, Machado-Joseph disease proteases; JAMMs, JAB1/MPN/Mov34 metalloproteases; TNBC, triple-negative breast cancer; GBM, glioblastoma; GSCs, glioblastoma stem cells; BCSCs, breast cancer stem cells; PDAC, pancreatic ductal adenocarcinoma; T-ALL, T-cell acute lymphoblastic leukemia; BCC, basal cell carcinoma; NB, neuroblastoma; CNS, central nervous system; MEFs, mouse embryonic fibroblasts; TGF-β, transforming growth factor-beta; TβRI/II/III, TGF-β receptor type I/II/III; Hh, Hedgehog; SHH, Sonic hedgehog; SMO, Smoothened; PTCH, Patched; Gli, glioma-associated oncogene homolog; NICD, Notch intracellular domain; CDH1, cadherin-1/E-cadherin; CDH2, cadherin-2/N-cadherin; VIM, vimentin; ZEB, zinc finger E-box-binding homeobox; SNAI1/2, Snail family transcriptional repressor 1/2; Twist1/2, Twist family bHLH transcription factor 1/2; ALDH1A3, aldehyde dehydrogenase 1 family member A3; HIF-1α, hypoxia-inducible factor-1 alpha; PYGL, glycogen phosphorylase L; PKM2, pyruvate kinase M2; HK2, hexokinase 2; GLUT1, glucose transporter 1; CHEK1/CHK1, checkpoint kinase 1; ID1, inhibitor of differentiation 1; FoxM1, forkhead box M1; SIRT1, sirtuin 1; DNMT1, DNA methyltransferase 1; APC, adenomatous polyposis coli; DVL, dishevelled; LRP6, low-density lipoprotein receptor-related protein; ESCC, esophageal squamous cell carcinoma.

## 4 DUBs regulate key signaling pathways mediating EMT and stemness maintenance

DUBs support the maintenance of CSC stemness and EMT phenotypes by modulating the stability, subcellular localization, and biological activity of components in multiple developmental signaling pathways. Among these, Wnt/β-catenin, Hh, Notch, and TGF-β signaling pathways are particularly important because they cooperatively regulate cell fate determination, phenotypic plasticity, invasion, and therapy resistance. In this section, we summarize current evidence regarding DUB-mediated regulation of major EMT- and stemness-associated pathways (Figure 2).

**Figure 2:**
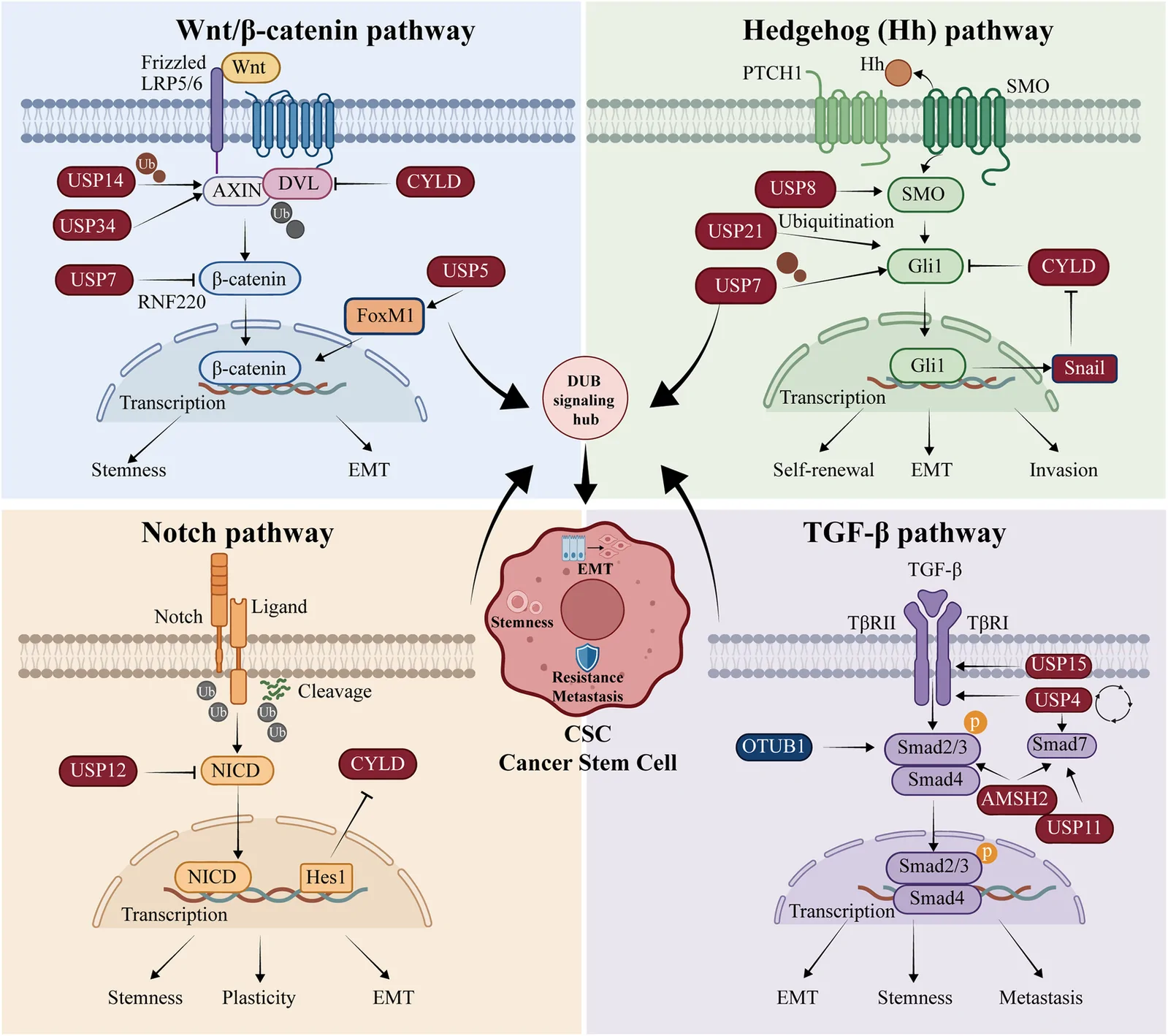
DUB-mediated integration of oncogenic signaling networks driving CSC plasticity, EMT, and therapeutic resistance. This figure illustrates how specific DUBs coordinate four major signaling cascades to maintain CSC stemness and therapeutic resistance. DUBs dynamically stabilize effectors and receptors across the Wnt/β-catenin (e.g., β-catenin/DVL via USP7/14), Hedgehog (e.g., SMO/Gli1 via USP8/21), Notch (e.g., NICD via USP12), and TGF-β (e.g., TβRI/Smads via USP15/OTUB1) pathways. Ultimately, these diverse DUB-regulated signaling axes converge to empower CSCs with enhanced self-renewal, metastatic capacity, and drug resistance.

### 4.1 DUBs and the Wnt/β-catenin signaling pathway

The canonical Wnt signaling pathway is a crucial regulator of EMT and tumor stem cell phenotypes. It regulates downstream gene expression through both the β-catenin-dependent canonical pathway and noncanonical signaling pathways (37). Aberrant activation of Wnt signaling is frequently observed during tumor progression (38), and the protein stability of β-catenin is dynamically regulated by the balance between E3 ubiquitin ligases and DUBs, thereby influencing Wnt signaling activity. Multiple E3 ubiquitin ligases are involved in modulating the stability of core Wnt pathway components, including β-catenin, APC, and DVL. Correspondingly, multiple DUBs functionally modulate these proteins through deubiquitination. For example, USP14 removes K63-linked polyubiquitin chains from DVL, reducing its ubiquitination level and activating Wnt/β-catenin signaling (39). Conversely, CYLD functions as a negative regulator of Wnt signaling, and its loss promotes β-catenin recruitment and pathway activation (40). Other DUBs exert regulatory effects at distinct nodes of this pathway. USP4 participates in Wnt signaling regulation by targeting NLK and TCF7L2, while USP34 has been reported to negatively regulate Wnt activity by maintaining the stability of AXIN1/2 and protecting these components of the degradation complex from ubiquitin-dependent degradation (41). TRABID is another Wnt-associated deubiquitinating enzyme that can deubiquitinate APC and modulate the stability of the β-catenin/TCF transcriptional complex (42). These findings indicate that DUB-mediated regulation extends beyond β-catenin itself, affecting multiple structural and regulatory layers of this pathway.

In fact, several DUBs, including USP9X, USP47 and USP4, have been reported to directly deubiquitinate β-catenin (43–45). DUB-mediated receptor regulation also affects cellular responses to Wnt signaling. USP8 (also known as UBPY) promotes the recycling of Frizzled (Fzd) to the plasma membrane by deubiquitinating Fzd, thereby maintaining receptor availability (46). Similarly, USP6 can deubiquitinate Fzd and low-density lipoprotein receptor-related protein 6 (LRP6), and inhibit the activity of the transmembrane E3 ubiquitin ligases RNF43/ZNRF3, thereby increasing the abundance of Fzd and LRP6 on the plasma membrane and enhancing cellular sensitivity to Wnt signals (47).

At the transcriptional level, DUBs can indirectly potentiate Wnt signaling by stabilizing β-catenin co-activators. For instance, USP5 promotes the accumulation of Forkhead box M1 (FoxM1) by removing ubiquitin chains from FoxM1 and preventing its degradation. Stable FoxM1 further facilitates the recruitment of β-catenin to Wnt target gene promoters, thereby enhancing transcriptional activation (48). In the absence of Wnt ligands, β-catenin is phosphorylated by the GSK3/AXIN/APC degradation complex, subsequently ubiquitinated by E3 ligases such as RNF220, and degraded by the proteasome. When Wnt ligand is present, this complex dissociates to protect β-catenin from degradation; meanwhile, USP7 interacts with RNF220 to promote β-catenin deubiquitination and enhance its stability, thereby further amplifying Wnt signal transduction (49). Importantly, β-catenin stabilized by DUBs not only drives EMT but also transcriptionally activates glycolytic enzymes such as PKM2, thereby coupling structural plasticity with metabolic adaptation. Crucially, the stabilizing effect of specific DUBs on β-catenin extends far beyond canonical EMT induction. Following nuclear translocation, stable β-catenin functions as a pivotal transcriptional co-activator, upregulating critical glycolytic transporters and enzymes such as GLUT1, PKM2, and LDHA (50). Therefore, by rescuing β-catenin from proteasomal degradation, DUBs essentially couple EMT-driven morphological changes with the high metabolic demands (e.g., aerobic glycolysis) required for cancer stem cell maintenance. Consequently, DUB-mediated stabilization of β-catenin not only promotes EMT but also enforces glycolytic reprogramming, linking developmental signaling to metabolic adaptation in CSCs.

### 4.2 DUBs and the Hh signaling pathway

The Hh pathway is another developmental signaling cascade closely associated with CSC stemness and EMT. Key components of the Hh pathway, including Patched (PTCH), Smoothened (SMO), and glioma-associated oncogene homolog (Gli) family proteins, are all subject to precise regulation by ubiquitination. SMO is a signal transducer of the Hh pathway localized at the plasma membrane: activated SMO transduces signals into the cytoplasm to initiate downstream signaling cascades, while polyubiquitination of SMO inhibits this signal transduction process, and DUB-catalyzed deubiquitination promotes SMO protein accumulation and subsequent Hh pathway activation. RNA interference (RNAi) screens in Drosophila and mammalian cell culture models revealed that USP8 directly deubiquitinates SMO, thereby driving SMO accumulation and downstream Hh signal activation. Notably, in the absence of Hh ligand, USP8 stabilizes SMO without altering its phosphorylation status, and SMO accumulation induced by USP8 remains under inhibitory regulation by PTCH. These findings suggest that USP8 exerts regulatory effects downstream of SMO phosphorylation events, yet remains constrained by upstream regulatory mechanisms of the Hh pathway (51).

Nuclear effector functions of the Hh signaling pathway are primarily mediated by the transcription factor Cubitus interruptus (Ci) in Drosophila and Gli family proteins in mammals. In Drosophila, Ci is regulated by two distinct ubiquitination-dependent pathways: in the absence of Hh ligand, Ci undergoes partial degradation mediated by the Slimb-Cullin1/β-transducin repeat-containing protein (β-TrCP) complex; upon Hh stimulation, Ci degradation mediated by the Hib-Cullin3-Roadkill complex finely tunes its transcriptional activity (52). In mammalian cells, Gli family proteins are similarly regulated by ubiquitination modifications, including ubiquitin binding catalyzed by the E3 ubiquitin ligase Itch (53).

USP7 competitively binds to Ci/Gli with its endogenous negative regulators Cos2 and Suppressor of Fused (Sufu), while Hh signaling promotes phosphorylation of Ci/Gli by enhancing the binding affinity between USP7 and Ci/Gli. By antagonizing Ci/Gli ubiquitination mediated by the Slimb-Cullin1/β-TrCP family E3 ubiquitin ligases, USP7 stabilizes Ci/Gli proteins and facilitates Hh signal transduction. Consistent with this regulatory mechanism, knockdown of USP7 in a cerebellar medulloblastoma model significantly inhibited the sonic hedgehog (SHH) pathway activity and reduced Gli protein expression levels (54).

USP21 has been identified as a positive regulator of Hh signaling. Genetic knockout of USP21 in mouse embryonic fibroblasts (MEFs) significantly attenuated Hh pathway activation induced by SMO agonists. In human embryonic kidney 293 T (HEK293 T) cells transfected with *GLI1*, USP21 depletion reduced *GLI1* promoter-driven reporter gene activity and modulated PKA-dependent Gli1 phosphorylation, collectively indicating that USP21 regulates Gli function through multiple distinct mechanisms. In addition to USP family DUBs, CYLD is also closely associated with tumorigenesis related to the Hh pathway. Downregulation of CYLD expression is frequently observed in basal cell carcinoma (BCC), a phenotype driven in part by the hyperactivation of Gli1/2. Mechanistically, aberrantly activated Gli1 transcriptionally upregulates the expression of the zinc-finger transcription factor Snail, which in turn transcriptionally represses *CYLD*, ultimately forming an oncogenic positive feedback loop (55). Taken together, DUBs exert multi-target and multi-layered regulation on the Hh pathway by modulating the stability and activity of the two signaling nodes, SMO and Ci/Gli. Importantly, the hyperactivation of the Hh/Gli axis maintained by these DUBs not only drives EMT but also triggers profound metabolic reprogramming. For instance, stable Gli1 can directly upregulate key metabolic enzymes such as hexokinase 2 (HK2) (56), suggesting that DUB-mediated amplification of Hh signaling equips CSCs with enhanced glycolytic capacity necessary for survival in nutrient-deprived tumor microenvironments. However, in pathological contexts such as BCC, the regulatory roles of other DUBs on PTCH and the entire Hh signaling cascade remain to be further elucidated. Therefore, Hh activation driven by DUBs combines EMT induction with enhanced glycolytic capacity, supporting the survival of CSCs in metabolically hostile niches.

### 4.3 DUBs and the Notch signaling pathway

The Notch signaling pathway is a highly conserved intercellular communication pathway that is widely active during embryonic development and in a range of adult tissues. It maintains tissue homeostasis by regulating cell differentiation, proliferation, and apoptosis, and also participates in the EMT process. The local microenvironment can influence Notch activity, and dysregulation of this pathway is closely associated with various diseases, including developmental disorders and cancers (57). Beyond its involvement in cellular plasticity, emerging evidence suggests that aberrant activation of Notch signaling may influence CSC metabolism, including c-Myc-dependent glutaminolysis and glycolysis, potentially supporting increased bioenergetic requirements. Consequently, DUBs that modulate the stability of the *Notch* intracellular domain (NICD) may function as context-dependent regulators linking Notch-associated EMT phenotypes to metabolic adaptation in CSCs.

Canonical Notch activation is initiated by the binding of membrane-bound ligands on donor cells to Notch receptors on adjacent recipient cells, which triggers sequential proteolytic cleavage and releases the NICD. NICD subsequently translocates to the nucleus, where it forms a transcriptional activation complex and induces the expression of downstream target genes. These signaling events are primarily mediated by DSL family ligands, including Delta, Serrate/Jagged, and LAG-2, although several non-canonical ligands have also been reported; their mechanisms of action are not fully understood (57). Among the DUBs implicated in this pathway, USP12 has emerged as a negative regulator of Notch signaling. Studies show that silencing USP12 impairs Notch lysosomal trafficking, leading to the accumulation of Notch receptors on the cell surface and enhanced pathway activation. Further biochemical evidence indicates that USP12 forms a complex with its cofactor UAF1 to negatively modulate Notch signaling by deubiquitinating the inactive form of the receptor (58). CYLD also participates in the regulation of Notch signaling and its downstream effects on EMT. In T-cell acute lymphoblastic leukemia (T-ALL) cell lines, it has been reported that the Notch-Hes1-CYLD axis can activate the IKK complex, thereby potentiating NF-*κ*B signaling (59). In addition, CYLD can negatively regulate the expression of the ligand *JAG2* through interaction with the E3 ubiquitin ligase MIB2. Loss of CYLD increases JAG2 levels and enhances Notch activity, subsequently promoting the upregulation of Snail and N-cadherin, thereby facilitating the acquisition of EMT-associated phenotypes (29, 60). Collectively, the Notch signaling pathway is tightly controlled by ligand-receptor interactions and ubiquitination modifications, and plays a context-dependent bidirectional role in tumorigenesis and EMT progression. Therefore, a deep understanding of how DUBs such as USP12 and CYLD modulate Notch signaling can not only provide mechanistic insights into their role in malignant progression but also lead to the discovery of new therapeutic targets and intervention strategies for cancer treatment.

### 4.4 Regulation of the TGF-β signaling pathway by DUBs and their role in EMT

As a ubiquitous cytokine, TGF-β regulates key processes such as cell growth, differentiation, and survival across multiple lineages, notably being recognized as a factor of the EMT program (61). Initiation of TGF-β signaling fundamentally relies on specific interactions between its ligand and trimeric high-affinity membrane receptors, namely TβRI, TβRII, and TβRIII (β-glycan). Upon receptor activation, TβRII and TβRI cooperate to induce phosphorylation of Smad2 and Smad3. Phosphorylated Smad2/3 proteins subsequently form complexes with Smad4 and translocate to the nucleus to drive transcription of target genes (61). In addition to the canonical Smad-dependent pathway, TβRI can also activate non-canonical signaling cascades, including Ras-related signaling mediated by adaptor proteins such as ShcA, Sos1, and Grb2 (62). Notably, the TGF-β signaling pathway, composed of components such as TβRI, TβRII, and Smads, is strictly regulated by the UPS (63). As a key component of this system, DUBs regulate TGF-β signaling activity and the EMT process by removing ubiquitination modifications from pathway components. USP4 is a structurally and functionally conserved DUB that modulates TGF-β signaling and EMT progression via multiple molecular mechanisms. On the one hand, USP4 directly deubiquitinates TβRI while inhibiting the ubiquitination and degradation of Smad7, and it can also maintain its own protein stability through self-deubiquitination (64). In a zebrafish xenograft model, USP4 enhances TGF-β signaling to promote invasion and metastasis of breast cancer cells, whereas USP4 knockdown significantly attenuates TGF-β-induced EMT in breast cancer cells. On the other hand, in colorectal cancer, USP4 interacts with PRL-3, which is a key stimulator of the EMT signaling pathway. Through deubiquitination, USP4 stabilizes PRL-3 expression and activates the Akt signaling pathway, ultimately leading to the downregulation of E-cadherin. High expression of PRL-3 has been confirmed to be closely associated with poor prognosis in cancer patients (65).

USP11 is another positive regulator of TGF-β signaling. It modulates TGF-β signaling by targeting the interaction between Smad7 and TβRI. Consistent with this mechanism, USP11 binds to Smad7 and masks its negative regulatory function while enhancing pathway activity through deubiquitination of TβRI. Knockdown of USP11 reduces TGF-β-induced Smad2/3 phosphorylation levels, thereby inhibiting the EMT process in mouse MECs (66). Similarly, using a multifunctional RNAi screening approach, Eichhorn et al. further identified USP15 as a key regulator of the TGF-β signaling pathway. USP15 interacts with the Smad3-Smurf3 complex and stabilizes TβRI through deubiquitination, thereby enhancing TGF-β signal transduction (67).

OTUB1 also modulates TGF-β signaling, albeit through a slightly different mechanism. It interacts with E2 ubiquitin-conjugating enzymes, blocking the transfer of ubiquitin from E2 to E3 ligases, thereby inhibiting the ubiquitination-mediated degradation of phosphorylated Smad2/3. In colorectal cancer cells SW480, Western blot analysis showed that OTUB1 knockdown increased the expression of ZEB1, β-catenin, N-cadherin, and VIM, while decreasing the level of E-cadherin, indicating that OTUB1 plays an important role in TGF-β-mediated gene transcription and cell migration (68). Furthermore, AMSH-2, a DUB containing an AMSH domain, is widely expressed in cells. Overexpression of AMSH-2 may activate the TGF-β promoter, and co-immunoprecipitation experiments have demonstrated interactions between AMSH-2 and both Smad7 and Smad2, suggesting a potential regulatory role in the TGF-β signaling pathway (69). Available evidence further suggests that prolonged DUB-associated TGF-β/SMAD signaling may influence not only the EMT program but also aspects of metabolic rewiring. For instance, activated SMAD complexes have been reported to affect mitochondrial dynamics and promote the Warburg effect, meeting the energy demands of migrating CSCs (70, 71). Collectively, these observations raise the possibility that DUBs associated with the TGF-β axis may contribute to the coordination of cellular plasticity and metabolic adaptation, although the underlying mechanisms and their relevance across tumor contexts require further investigation.

## 5 Crosstalk between DUBs and transcription factors: a bidirectional network regulating EMT and stemness

DUBs and transcription factors may form a multilayered, bidirectional regulatory network involved in the EMT process, CSC-associated properties, chemoresistance, and metastatic potential. Extensive evidence suggests that EMT and its associated transcription factors are central drivers of tumor cells acquiring CSC-like properties (72). For example, the key EMT transcription factor Twist1 can simultaneously mediate EMT in breast cancer, maintain CSC self-renewal, promote tumor metastasis, and reduce the sensitivity of cancer cells to chemotherapeutic agents (73). Notably, Twist1 is also a key driver of tumor metabolic reprogramming, particularly in promoting the Warburg effect. Thus, DUB-mediated stabilization of these transcription factors may provide one mechanism through which phenotypic plasticity and metabolic adaptation are coordinately regulated. By modulating the ubiquitination and stability of EMT-associated and stemness-related transcription factors, DUBs may function as context-dependent regulatory nodes connecting EMT, tumor stemness, and therapeutic resistance.

Among DUBs that positively regulate transcription factor stability, members of the USP family are particularly prominent. USP29 stabilizes the Twist1 protein through deubiquitination, thereby promoting the progression of triple-negative breast cancer (TNBC) (73). USP4 simultaneously stabilizes β-catenin and Twist1 in colorectal cancer cells, enhancing tumor stemness and inducing chemoresistance (74). UCHL3 maintains the tumorigenic potential and stemness features of hepatocellular carcinoma (HCC) by enhancing the stability of β-catenin (75). USP21 specifically removes K48-linked ubiquitin chains from the stemness transcription factor Nanog, preventing its proteasomal degradation and stabilizing the Nanog protein. This mechanism not only helps maintain embryonic stem cell pluripotency but also contributes to the regulation of pancreatic cancer stemness (76). In addition, USP22 is frequently overexpressed in lung cancer and other malignancies and is widely regarded as a marker associated with tumor stem cells; in gastric cancer, USP22 also maintains and enhances tumor stemness by stabilizing BMI1 (77).

In colon cancer tissues, OTUB2 expression is significantly elevated. OTUB2 knockdown attenuates stemness characteristics, increases sensitivity to oxaliplatin, inhibits EMT, and ultimately reduces metastatic potential. Mechanistically, OTUB2 specifically deubiquitinates the transcription factor SP1, inhibiting its K48-linked ubiquitination-mediated degradation and thereby maintaining its stability. *SP1* subsequently binds to the 1,822–1,830 region of the *GINS1* promoter and enhances its transcriptional activity. The resulting OTUB2-SP1-GINS1 axis plays a key role in driving colon cancer stemness, chemoresistance, and EMT (78). USP14 interacts directly with the stemness transcription factor SOX2 through its ubiquitin carboxyl-terminal hydrolase domain. USP14 knockdown increases polyubiquitination of SOX2 and reduces its stability, thereby impairing the maintenance of CSC markers (79). USP7 interacts with the basal transcription factor BTF3 and upregulates its protein level, thereby enhancing the EMT phenotype and stem cell-like characteristics of HCC cells (80). Moreover, USP28 regulates the stability of the transcription factor c-Myc, thereby inhibiting the growth of c-Myc-overexpressing neuroblastoma (NB) (81). Given that c-Myc is a key regulator of aerobic glycolysis and glutamine metabolism, DUB-mediated stabilization of c-Myc directly links the maintenance of rapid CSC proliferation with its required metabolic flexibility.

Notably, the interaction between DUBs and transcription factors is not limited to unidirectional control; they frequently participate in bidirectional feedback loops that finely tune CSC properties. For instance, in glioma stem cells, the key transcription factor E2F1 directly transactivates the expression of *USP5*. Conversely, USP5 maintains the post-translational stability of the stemness factor OCT4, thereby determining the self-renewal and tumorigenic capacity of these cells (82). Similarly, in ovarian cancer, c-Jun binds to the enhancer region of *OTUD1* to promote its transcription. Elevated OTUD1 subsequently forms aggresomes, recruits the ASK1 kinase, and hyperactivates the JNK/c-Jun signaling cascade. This c-Jun-OTUD1 positive feedback loop is critical for maintaining the self-renewal and tumorigenicity of ovarian cancer stem cells (OCSCs) (83). Furthermore, the transcription factor AP-2 can directly drive the transcription of *USP22*. Clinically, a positive correlation between AP-2 and USP22 has been observed in lung cancer tissues, indicating that this regulator*y* axis is a fundamental driver of lung adenocarcinoma progression (84).

The EMT-TFs stabilized by these interconnected DUB networks may contribute not only to cellular dedifferentiation but also to metabolic reprogramming. For instance, DUB-stabilized SNAI1 and Twist1 have been shown to directly inhibit mitochondrial OXPHOS genes while cooperatively transactivating key glycolytic enzymes (85–87). Consequently, DUBs situated within these transcriptional circuits serve as molecular hubs. By simultaneously conferring mesenchymal traits, amplifying stemness, and rewiring metabolic fluxes, they could contribute to CSC plasticity and facilitate adaptation to stressful tumor microenvironments, potentially reducing sensitivity to conventional therapies.

## 6 The orchestration of metabolic rewiring by DUBs in CSCs

Rather than merely occurring as a passive consequence of EMT, metabolic alterations may also be subject to regulation by DUBs, including through changes in the stability of metabolic enzymes, transporters, and other metabolic regulators. By removing ubiquitin chains from specific metabolic proteins, DUBs may influence the metabolism of glucose, lipids, and amino acids, potentially contributing to the bioenergetic and biosynthetic requirements associated with phenotypic plasticity in CSCs. In addition, available evidence suggests that these metabolic programs may interact with stemness- and EMT-related signaling networks, potentially establishing reciprocal regulatory relationships associated with tumor progression and treatment resistance.

### 6.1 Reprogramming of glucose metabolism: the Warburg effect

Aerobic glycolysis, also known as the Warburg effect, is one of the most prominent metabolic features of CSCs. Although OXPHOS remains important in certain tumor contexts, many CSC populations preferentially rely on glycolysis to rapidly generate ATP and metabolic intermediates while limiting the accumulation of ROS (88). This metabolic phenotype is not only a result of oncogenic signaling but is also actively maintained by DUBs that stabilize rate-limiting glycolytic enzymes and glucose transport machinery (89).

Among the best-characterized mechanisms, multiple DUBs have been shown to regulate the abundance and activity of glycolytic enzymes such as phosphofructokinase (PFK), pyruvate kinase M2 (PKM2), and lactate dehydrogenase A (LDHA). PKM2 is frequently overexpressed in cancer and serves as a core regulator of the Warburg effect. In various types of cancer, multiple DUBs modulate the activity and stability of PKM2 by modulating its ubiquitination status. In colorectal cancer, OTUB2 inhibits the ubiquitination of PKM2 and enhances its enzymatic activity (89). In ovarian cancer, PSMD14 interacts directly with PKM2, reducing its ubiquitination level to maintain protein stability (90). In addition, USP20 (91) and USP7 (92) can also bind to PKM2 and regulate its ubiquitination homeostasis. In gastric cancer, USP4 interacts with PKM2 and catalyzes its deubiquitination; high expression of USP4 significantly promotes the proliferation, glucose uptake, and lactate production of gastric cancer cells (93). These findings suggest that DUBs do not merely act on isolated metabolic enzymes but integrate metabolic flux with stemness-associated transcriptional programs.

Emerging evidence supports the concept that DUBs can directly stabilize metabolic enzymes. In non-small cell lung cancer (NSCLC), OTUB2 promotes malignant progression and metabolic reprogramming through dual regulation. On the one hand, OTUB2 enhances proliferation, colony formation, migration, and invasion, thereby augmenting the malignant phenotype. On the other hand, it activates the Warburg effect and the AKT/mTOR signaling pathway, a classic pro-tumorigenic pathway that centrally regulates cellular growth and metabolic homeostasis. Mechanistically, OTUB2 directly binds to U2AF2 and removes its ubiquitin modifications, thereby stabilizing U2AF2 and preventing its proteasomal degradation. This regulatory mechanism is critical for OTUB2-driven NSCLC progression (94). Since CSCs are typically located in hypoxic and nutrient-deprived niches, the ability of DUBs to maintain the stability of glycolytic enzymes may provide a selective survival advantage by sustaining ATP production and the generation of biosynthetic precursors. Moreover, lactate accumulation resulting from enhanced glycolysis can acidify the tumor microenvironment, thereby promoting invasion, immune evasion, and EMT. Therefore, DUB-driven glycolytic rewiring serves as a metabolic axis maintaining CSC plasticity.

### 6.2 DUBs and amino acid metabolism in CSCs

Amino acid metabolism, particularly glutamine metabolic reprogramming, is a key mechanism by which CSCs adapt to metabolic demands and sustain unlimited proliferation. Glutamine not only serves as a primary carbon and nitrogen source for nucleotide synthesis, glutathione production, and the tricarboxylic acid (TCA) cycle but also synergizes with enhanced antioxidant defense mechanisms to help cells resist oxidative stress and therapeutic damage. As important regulators of the UPS, DUBs, including USP13, USP9X, USP15, USP29, and USP39, can finely tune the synthesis, catabolism, and transport of glutamine by stabilizing relevant transcription factors, metabolic enzymes, and transporters. This reshapes the metabolic flux of α-ketoglutarate (α-KG), nucleotides, and lipids, promoting tumor cell survival, proliferation, and the maintenance of CSC stemness.

DUBs play crucial roles in regulating glutamine synthesis, catabolism, and transport across various cancers. In ovarian cancer, USP13 acts as a central driver of glutamine metabolic reprogramming by deubiquitinating and upregulating ATP citrate lyase and α-ketoglutarate dehydrogenase (α-KGDH), thereby coupling glutamine catabolism with mitochondrial respiration and fatty acid synthesis to supply energy and biosynthetic precursors to cells (95). Similarly, in NB, USP29 links glutamine catabolism to nucleotide synthesis, supporting cell proliferation; loss of USP29 disrupts the metabolism of glycolytic and nucleotide biosynthesis intermediates (96). In GBM, glutamine synthesis depends on the coordinated regulation of USP9X and HDAC6, where USP9X removes K48-linked ubiquitin chains from glutamine synthetase (GS) to stabilize the protein, while HDAC6 enhances its enzymatic activity and stability through deacetylation. Together, they maintain glutamine production under conditions of glutamine deprivation (97). In multiple myeloma, USP15 antagonizes CRL4(CRBN) E3 ligase-mediated GS ubiquitination, preventing its degradation; notably, USP15 is highly expressed in immunomodulatory drug (IMiD)-resistant MM cells, and silencing USP15 restores sensitivity to lenalidomide, indicating its significant role in metabolic regulation and drug resistance (98).

DUBs also regulate glutamine transport. Glutamine enters cells through transporters such as ASCT2/SLC1A5, is subsequently converted to glutamate by mitochondrial glutaminase, and is further metabolized into α-KG, thereby supplying rapidly proliferating cancer cells with ATP, biosynthetic precursors, and reducing equivalents (99). In NSCLC, the USP39-MRPL35-SLC7A5 axis represents a key pathway controlling glutamine uptake: USP39 stabilizes MRPL35 through deubiquitination, which in turn upregulates the glutamine transporter SLC7A5. Increased *SLC7A5* expression significantly enhances glutamine uptake and metabolism, supporting NSCLC proliferation and invasion (100). Beyond glutamine metabolism, amino acid-dependent redox regulation is also closely associated with CSC survival and therapeutic resistance. However, direct evidence regarding the role of DUBs in regulating serine/glycine-derived one-carbon metabolism in CSCs remains limited. Current evidence more strongly supports the role of DUBs in amino acid-associated redox control, particularly through the regulation of the cystine/glutamate antiporter SLC7A11 and ferroptosis sensitivity. For example, BAP1 suppresses SLC7A11 expression through H2A deubiquitination at the SLC7A11 promoter, thereby limiting cystine uptake and rendering cancer cells sensitive to ferroptosis (101). Conversely, OTUB1 stabilizes SLC7A11, promoting cystine uptake, glutathione biosynthesis, and ferroptosis resistance (102). These findings indicate that DUBs regulate amino acid-dependent redox homeostasis through specific mechanisms. Whether specific DUB-substrate pairs directly control serine/glycine pathway enzymes, such as phosphoserine aminotransferase 1, phosphoserine phosphatase, or methylenetetrahydrofolate dehydrogenase family, in CSCs remains an important area for future research.

### 6.3 DUBs and lipid metabolic rewiring in CSCs

Reprogramming of cholesterol metabolism is a key characteristic enabling cancer cells to meet biosynthetic demands and maintain CSC stemness. Aberrant activation of key pathways—such as the mevalonate pathway and pathways regulating cholesterol uptake and synthesis—is closely associated with tumor progression. Recent studies have confirmed that DUBs can reprogram cholesterol metabolic flux in cancer cells by stabilizing key regulators of cholesterol metabolism and modulating pathway activities, thereby influencing tumor proliferation, drug resistance, and the maintenance of CSC stemness. These findings provide new molecular mechanisms and potential targets for metabolism-based cancer therapies.

DUBs regulate cholesterol uptake and synthesis pathways. During lipid uptake, ubiquitin carboxyl-terminal hydrolase 1 (UCHL1) stabilizes the scavenger receptor CD36 through deubiquitination. CD36 not only mediates the uptake of oxidized low-density lipoprotein (oxLDL) to promote foam cell formation—a key event in atherosclerosis—but also regulates lipid uptake, including cholesteryl esters, in various cancers (103). Studies have shown that knockdown or inhibition of UCHL1 increases K48-linked ubiquitination of CD36 and promotes its proteasomal degradation, thereby reducing oxLDL uptake and foam cell formation (103). Although its direct role in cancer has not yet been fully established, CD36-mediated metabolic reprogramming is crucial for energy supply in tumor cells, suggesting that UCHL1 may regulate cholesterol-related lipid metabolism in tumor cells through CD36 and indirectly participate in EMT.

BAP1 is a component of the polycomb repressive deubiquitinase complex (PR-DUB) and is frequently lost in malignant mesothelioma and various other cancers. BAP1 can indirectly regulate cholesterol biosynthesis via PRC2, and its loss creates a therapeutic window known as “PRC2-dependent cholesterol metabolism vulnerability”. Meanwhile, BAP1 deficiency leads to significant upregulation of cholesterol biosynthesis-related proteins, including mevalonate pathway enzymes, in the liver, accompanied by hypercholesterolemia and hepatic lipid depletion (104). As BAP1 is a classic tumor suppressor gene, dysregulation of its control over cholesterol synthesis may establish a metabolic basis for hepatic tumorigenesis, indicating that BAP1-mediated cholesterol metabolic reprogramming could be a potential driver of early carcinogenesis (104).

USP28 is highly expressed in squamous carcinomas such as lung squamous cell carcinoma and shows a significant positive correlation with protein levels of SREBP2, a key transcription factor regulating cholesterol synthesis, and is associated with poor patient prognosis (105). Mechanistically, USP28 directly binds the mature form of SREBP2 and stabilizes it by removing its K48-linked ubiquitin chains, thereby activating the mevalonate pathway. This pathway not only synthesizes cholesterol but also produces isoprenylation substrates such as geranylgeranyl pyrophosphate, providing essential protein modification substrates for cancer cell proliferation and CSC stemness maintenance (105). Functional studies show that USP28 knockdown reduces mevalonate pathway flux and increases cancer cell sensitivity to statins, which are inhibitors of this pathway, while supplementation with geranylgeranyl pyrophosphate can reverse the tumor-suppressive effects of USP28 deficiency (105). Furthermore, combined treatment with dual USP28/25 inhibitors and statins significantly inhibits the survival of squamous cell carcinoma, confirming USP28's status as a critical therapeutic target in the cholesterol metabolism-cancer growth axis (105).

The combined modulation of DUB activity and lipid metabolism has been explored as a potential therapeutic approach. In TNBC, USP14 promotes tumor cell survival by stabilizing the anti-apoptotic protein Survivin. The cholesterol metabolism inhibitor lovastatin was shown to interfere with the interaction between USP14 and Survivin, enhances Survivin's ubiquitination-mediated degradation, and induces apoptosis in TNBC cells (106). Moreover, combined use of USP14 inhibitors such as IU1 and lovastatin synergistically inhibits colony formation in TNBC cells, demonstrating that drugs targeting cholesterol metabolism can enhance antitumor efficacy by interfering with DUB-substrate interactions (106). BRCC3 (BRCA1/BRCA2-containing complex subunit 3) is another member of the DUB family, which has been implicated in Tet2-deficient clonal hematopoiesis. It remodels the inflammatory microenvironment by regulating cholesterol metabolites, thereby promoting tumor-associated pathologies such as atherosclerosis and potential hematological malignancies. However, its detailed mechanisms and connections to EMT and CSC remain to be further elucidated.

### 6.4 DUBs regulate cancer stem cell survival through ferroptosis

Ferroptosis is an iron-dependent, lipid peroxidation-driven form of regulated cell death. Its dysregulation is profoundly implicated in cancer progression, CSC maintenance, and therapy resistance (107). By modulating the ubiquitination homeostasis of key regulators in oxidative and metabolic pathways, DUBs serve as critical nodes connecting ferroptosis evasion and CSC stemness. Recent studies suggest that DUBs may contribute to the association between metabolic adaptation and ferroptosis resistance, although their specific relevance to CSC survival remains to be established. For instance, in TNBC, the USP24-DHODH axis has been implicated in the regulation of ferroptosis sensitivity (108). Mechanistically, *USP24* stabilizes dihydroorotate dehydrogenase (DHODH), a mitochondrial enzyme critical for maintaining redox balance and detoxifying lipid peroxides. By retaining DHODH, USP24 provides a direct mechanism for CSC tolerance to severe treatments and oxidative stress. Beyond direct redox regulation, DUB-driven metabolic rewiring collaborates closely with epigenetic and post-translational networks to enhance stress tolerance. In gastric cancer, the PSMD14-PFKFB2-H3K27 lactylation cascade demonstrates how PSMD14-stabilized PFKFB2 enhances glycolytic flux (109). Accumulated lactate reshapes the tumor metabolic microenvironment and drives H3K27 lactylation to activate stemness-associated transcription. Strikingly, this lactate-rich microenvironment can further trigger feed-forward loops to enhance ferroptosis resistance. A recent study on intrahepatic cholangiocarcinoma (ICC) revealed that L-lactate directly promotes K100 lactylation of PSMD14, shielding the DUB from proteasome-mediated degradation (110). Stable PSMD14 subsequently interacts with the glycolytic enzyme ENO1, removing its K63-linked ubiquitin chains and preventing its lysosomal degradation. This L-lactate/PSMD14/ENO1 positive feedback loop not only promotes sustained lactate production but also drives profound ferroptosis resistance, ultimately compromising the efficacy of anti-PD-1 immunotherapy. Thus, DUBs may link ferroptosis resistance with metabolic, epigenetic, and immune remodeling, potentially contributing to CSC plasticity.

Basic insights into ferroptosis regulation in stem cells also derive from normal physiological models, which typically share vulnerabilities with CSCs. For example, the histone deubiquitinase MYSM1 is critical for protecting normal hematopoietic stem cells (HSCs) from ferroptosis (111). MYSM1 deficiency severely impairs protein synthesis, rendering HSCs highly susceptible to lipid peroxidation attacks, ultimately leading to bone marrow failure. Remarkably, in MYSM1-deficient models, pharmacological blockade of ferroptosis can completely rescue the HSC population and their function even without correcting the underlying protein synthesis defect (111). Because protein synthesis has been linked to ferroptosis sensitivity in human stem cells, elevated *MYSM1* activity observed in some malignancies may also reduce ferroptosis sensitivity in CSCs, although this possibility remains to be tested. These findings suggest a potential association between histone deubiquitination and stem-cell survival under ferroptotic stress.

In solid tumor contexts, specific DUBs actively dismantle ferroptosis cascades to preserve CSC characteristics, providing viable therapeutic targets. In HCC, UCHL3 orchestrates ferroptosis evasion through the UCHL3-β-catenin axis (75). Elevated UCHL3 removes K48-linked polyubiquitin chains from the ARM domain of β-catenin, preventing its proteasomal degradation. This stabilization not only drives HCC proliferation and stemness but also actively suppresses ferroptosis by attenuating the accumulation of ROS and malondialdehyde (MDA). Consequently, targeting UCHL3 with TCID promotes β-catenin degradation, strongly triggers ferroptosis, and restricts tumor growth, underscoring this axis as a key vulnerability in HCC (75).

Similarly, targeting DUBs can simultaneously activate multiple cell death pathways to overcome CSC resistance. In ovarian cancer, elevated USP2 is associated with poor prognosis, and its pharmacological blockade reveals a multi-layered regulation of tumor cell death. The small-molecule inhibitor ML364 of USP2 simultaneously triggers apoptosis (via downregulation of cyclin D1 and PARP cleavage) and ferroptosis (via a sharp increase in intracellular ROS levels) (112). The antitumor efficacy of ML364 depends on the ferroptosis pathway, confirming the role of USP2 as a dual-modulator of apoptosis and ferroptosis. Collectively, these studies indicate that targeting DUB-associated regulation of ferroptosis may warrant further investigation as a potential approach for reducing CSC survival (Figure 3).

**Figure 3:**
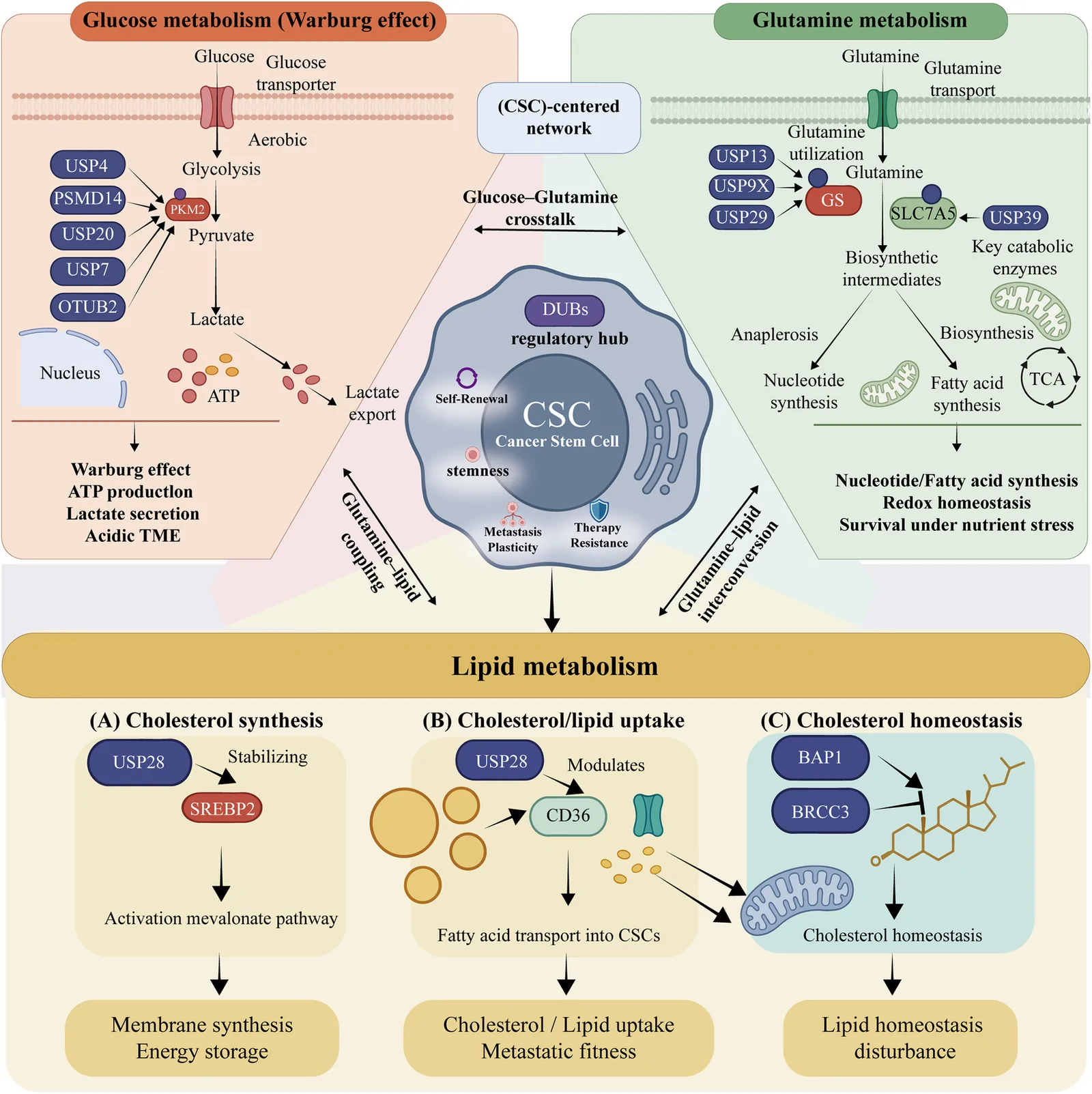
DUB-mediated metabolic reprogramming in the maintenance of cancer stem cell stemness. DUBs reshape the metabolic landscape of CSCs to support their energetic and biosynthetic demands. This figure details three interconnected metabolic axes: Glucose metabolism (Warburg effect)—DUBs such as USP4, PSMD14, USP20, USP7, and OTUB2 stabilize PKM2 and LDHA, promoting aerobic glycolysis and lactate production. Glutamine metabolism—USP13, USP9X, USP29 and USP39 regulate GS, SLC7A5, and key catabolic enzymes, fueling nucleotide and fatty acid synthesis. Lipid metabolism—USP28 stabilizes SREBP2 to drive the mevalonate pathway; UCHL1 modulates CD36-mediated lipid uptake; BAP1 and BRCC3 influence cholesterol homeostasis. By controlling these metabolic programs, DUBs reinforce CSC stemness, therapy resistance, and metastatic potential.

## 7 Bidirectional interactions between epigenetic regulation and metabolites

Cancer stem cell plasticity is regulated not only by transcription factors and developmental signaling pathways but also by the dynamic interplay between cellular metabolism and the epigenetic machinery. Metabolic pathways generate substrates and cofactors required for chromatin-modifying enzymes, thereby enabling nutrient availability, oxygen tension, mitochondrial function, and redox status to influence DNA methylation and histone modifications (113). Conversely, epigenetic regulators alter the expression of metabolic enzymes and transporters, generating reciprocal feedback loops that stabilize stem-like, mesenchymal, and therapy-resistant cellular states (114). DUBs reside at the nexus of this network, as they regulate both the stability or activity of epigenetic regulators and the stability or activity of metabolic signaling proteins (115).

### 7.1 Histone modifications and DNA methylation

Beyond transcriptome remodeling, epigenetic dysregulation—including DNA methylation and histone post-translational modifications (HPTMs)—has been extensively demonstrated to be closely associated with cancer initiation and progression (116). Under physiological conditions, these epigenetic mechanisms precisely regulate DNA packaging and chromatin formation, thereby maintaining genomic stability. In cancer, however, the dysregulation of these processes disrupts this balance and drives malignant transformation. Notably, the reversibility of epigenetic regulation makes it a focal point in current efforts to overcome cancer drug resistance (116). Within the context of CSC biology, epigenetic regulation is particularly important because it provides a reversible mechanism that enables cancer cells to dynamically transition between stem-like and differentiated states, adapt to microenvironmental pressures, and acquire therapy-tolerant phenotypes. Thus, DUB-mediated DNA methylation and histone modifications should be viewed not merely as general features of tumor progression but as critical regulatory layers maintaining CSC and phenotypic plasticity. Importantly, these epigenetic processes are tightly coupled to cellular metabolism, as many chromatin-modifying enzymes require metabolites as substrates or cofactors (117). Therefore, metabolic rewiring can directly influence chromatin accessibility and transcriptional plasticity during EMT-associated CSC maintenance. In some cancers, the expression of the tumor suppressor CYLD is suppressed by epigenetic mechanisms, including DNA methylation and histone deacetylase (HDAC)-mediated histone deacetylation. For example, in gastric adenocarcinoma, hypermethylation of the *CYLD* promoter region significantly downregulates the expression of *CYLD* mRNA and protein, thereby eliminating its tumor-suppressive function (118).

DNMT1 is a core epigenetic regulator responsible for maintaining DNA methylation homeostasis and plays a critical role in preserving parental DNA methylation patterns after replication. USP7 serves as an epigenetic modulator of DNMT1-dependent DNA methylation, exhibiting dual functions in both maintenance and *de novo* DNA methylation; *in vitro* assays demonstrate that USP7 can enhance the methyltransferase activity of DNMT1 by approximately two-fold (119). Meanwhile, evidence suggests that this epigenetic regulation is closely linked to EMT-metabolic crosstalk. Specifically, USP7-stabilized DNMT1 can silence the promoters of epithelial genes, such as CDH1 which encodes E-cadherin, thereby promoting the EMT process. Simultaneously, DNMT1-mediated hypermethylation has been shown to repress metabolic genes, including *FBP1*, which encodes fructose-1,6-bisphosphatase 1, promoting a metabolic shift toward aerobic glycolysis (120). Through this coordinated epigenetic mechanism, the USP7-DNMT1 axis acts as a node that links mesenchymal traits with the metabolic rewiring supporting CSC migration and survival.

In hepatoblastoma (HB), USP7 forms a novel ternary protein complex with UHRF1 and DNMT1 (121). This complex specifically binds to the promoters of key tumor suppressor genes in HB—including *HHIP* (Hedgehog-interacting protein), *IGFBP3* (insulin-like growth factor binding protein 3), and *SFRP1* (secreted frizzled-related protein 1)—and transcriptionally represses these genes through the cooperative regulation of DNA methylation (promoter hypermethylation) and histone modifications (such as H3K27 deacetylation), thereby promoting the malignant progression of HB (121). In addition, USP10 also plays an important regulatory role in NSCLC. The protein stability of HDAC7 is directly maintained by USP10-mediated deubiquitination, which protects it from degradation. Once stabilized, HDAC7 orchestrates the nuclear accumulation of β-catenin, enabling it to interact with TCF4 to drive transcriptional activation. The β-catenin–TCF4 complex activates the transcription of fibroblast growth factor 18 (*FGF18*), ultimately promoting NSCLC proliferation, invasion, and metastasis through FGF18-mediated signaling pathways (122). Inhibition of USP10 significantly accelerates HDAC7 degradation and impairs the growth and migratory capacity of NSCLC (122). These findings further indicate that DUB-mediated epigenetic regulation is functionally linked to signaling pathways in CSC biology, including the Wnt/β-catenin signaling pathway and the transcriptional repression of tumor-suppressive programs. More broadly, by stabilizing epigenetic modifiers or remodeling chromatin-associated complexes, DUBs may promote stemness maintenance, EMT-associated plasticity, and adaptation to therapeutic stress, rather than merely supporting bulk tumor growth.

### 7.2 Histone ubiquitination and chromatin plasticity

Histone ubiquitination is a key component of epigenetic regulation, and its dynamic balance is maintained jointly by ubiquitin ligases and DUBs. DUBs regulate gene expression associated with CSC stemness by specifically removing ubiquitin chains from histones such as H2A, H2B, H3, and H4. Ultimately, this process affects biological characteristics such as self-renewal, differentiation potential, drug resistance, and metastatic ability. Importantly, chromatin plasticity is a central determinant of CSC adaptability, because it enables rapid and reversible reprogramming of lineage identity, stress responses, and invasive behavior. From this perspective, histone-directed DUB activity is not only an epigenetic housekeeping process but also a mechanism that may directly influence whether tumor cells retain stem-like features or transition into therapy-persistent and metastasis-competent states. In addition, USP38 regulates the deacetylase activity of HDAC1 through deubiquitination, participating in the DDR, particularly non-homologous end joining (NHEJ) repair, and playing a critical role in maintaining genomic integrity and cancer cell resistance to genotoxic stress (123). Given the close association between efficient DNA damage repair and the survival of CSCs following chemotherapy or radiotherapy, this chromatin-centered DUB function may also contribute to the persistence of therapy-resistant CSC populations under therapeutic pressure.

### 7.3 Metabolite-dependent epigenetic regulation and metabolic-epigenetic feedback

Cellular metabolism directly shapes the epigenetic state by controlling the availability of substrates and cofactors required by chromatin-modifying enzymes. Acetyl-CoA supplies acetyl groups for histone acetyltransferases (124), while S-adenosylmethionine (SAM) serves as a methyl donor for DNA and histone methyltransferases (125). Conversely, α-KG is an essential cofactor for ten-eleven translocation (TET) DNA demethylases and Jumonji C-domain histone demethylases (126, 127). The oncometabolite 2-hydroxyglutarate (2-HG), along with accumulated succinate and fumarate, can competitively inhibit α-KG-dependent dioxygenases, thereby promoting DNA and histone hypermethylation (126, 128). In parallel, the NAD+/NADH balance influences NAD⁺-dependent sirtuin deacetylases (129). These represent the precise biochemical mechanisms by which metabolic reprogramming alters chromatin accessibility, transcriptional plasticity, and the maintenance of EMT-associated CSC states.

This relationship is bidirectional rather than linear. Epigenetic regulators control the transcription of glucose transporters, mitochondrial enzymes, lipid-metabolic enzymes, and amino acid-metabolic pathways, while the resulting metabolic changes modify intracellular levels of acetyl-CoA, SAM, α-KG, NAD+, and oncometabolites, feeding back to act on chromatin-modifying enzymes (114). DUBs may connect these processes by regulating the stability or activity of epigenetic and metabolic effectors. For example, the USP28-LSD1 and USP22-SIRT1 axes discussed above illustrate how DUB-dependent stabilization of chromatin regulators maintains stemness and EMT-associated transcriptional programs (19, 34). Similarly, DUB-mediated stabilization of β-catenin, c-Myc, HIF-1α-related signaling components, or metabolic enzymes may reshape glycolysis, glutaminolysis, and mitochondrial metabolism, indirectly changing the metabolite pool available for epigenetic reactions (115). Therefore, DUBs should be viewed as context-dependent modulatorsof metabolic-epigenetic feedback rather than isolated regulators of histone ubiquitination.

However, it is necessary to distinguish these indirect regulatory links from direct metabolite-dependent control of DUB catalytic activity. Compared to the established roles of acetyl-CoA, SAM, α-KG, 2-HG, succinate, fumarate, and NAD + in specific chromatin-modifying reactions, there is currently insufficient evidence that these metabolites directly bind to DUBs or regulate their catalytic domains under physiological conditions. Changes in DUB abundance or activity following metabolic perturbation may result from alterations in transcription, localization, post-translational modifications, redox status, or substrate availability. Thus, demonstrating direct metabolite-DUB regulation requires pure enzyme kinetic studies, direct-binding assays, structural studies, and cellular rescue experiments using catalytic or metabolite-binding-deficient mutants. Until such evidence is obtained, direct metabolic control of DUB activity should be regarded as a plausible but unproven mechanism in CSC plasticity.

## 8 Therapeutic potential of targeting DUBs

DUBs have emerged as highly attractive therapeutic targets in oncology due to their critical roles in stabilizing stemness-associated oncogenic transcription factors, metabolic regulators, and signaling components. Unlike conventional cytotoxic agents that primarily eliminate rapidly proliferating bulk tumor cells, therapies targeting DUBs hold promise for disrupting molecular circuits maintaining CSC homeostasis, EMT, metabolic plasticity, and treatment resistance. Given that many DUBs occupy key nodes downstream of multiple signaling pathways, pharmacological inhibition of these enzymes could simultaneously suppress tumor stemness, diminish metastatic capacity, and re-sensitize resistant tumors to conventional therapies. Importantly, DUBs are also enzymatically druggable. Numerous members of the USP, UCH, OTU, and JAMM families possess catalytic domains or ubiquitin-binding pockets suitable for small-molecule targeting. Over the past decade, several DUB inhibitors have entered preclinical development, with a few advancing to early clinical evaluation. Although no anti-cancer drugs specifically targeting DUBs have been approved for clinical use yet, accumulating evidence strongly supports the translational potential of this therapeutic strategy, particularly in combination regimens aimed at overcoming chemoresistance, radioresistance, and adaptive metabolic reprogramming. Beyond conventional catalytic inhibitors, an emerging and conceptually distinct strategy involves the development of deubiquitinase-targeting chimeras (DUBTACs), a class of molecules that extends the therapeutic scope from DUB inhibition to DUB-mediated selective protein stabilization. Unlike small molecules that globally inhibit DUB activity or block substrate binding, DUBTACs are heterobifunctional molecules designed to recruit selected DUBs to target proteins, thereby promoting deubiquitination of the target and protecting it from proteasomal degradation. This modality is particularly compelling in cancer and stemness research, as it enables the restoration of proteins with tumor-suppressive, pro-differentiation, or sensitization functions, which are typically unstable in aggressive tumors. Recent studies have established proof-of-concept DUBTAC platforms based on OTUB1, USP7, and USP28, highlighting the feasibility of selectively repurposing DUBs as a therapeutic strategy. Rather than simply shutting down DUB activity, DUBTACs offer the possibility of substrate-directed reprogramming of ubiquitin-dependent signaling pathways. In microenvironments where indiscriminate DUB inhibition is limited by toxicity or pathway redundancy, this approach could potentially improve target selectivity and expand the therapeutic window, although these benefits require further preclinical and clinical validation.

Among existing compounds, inhibitors targeting proteasome-associated DUBs and USP family members have attracted the most attention. b-AP15 is one of the earliest reported small-molecule inhibitors targeting the proteasome-associated DUBs *USP14* and UCHL5/UCH37. By blocking deubiquitination at the 19S regulatory particle of the proteasome, b-AP15 induces the accumulation of polyubiquitinated proteins, proteotoxic stress, endoplasmic reticulum stress, and oxidative damage, ultimately triggering apoptotic cell death (130). In models of multiple myeloma and various solid tumors, b-AP15 has demonstrated significant antitumor activity, including efficacy against bortezomib-resistant cells, suggesting that DUB inhibition may overcome resistance mechanisms associated with classical proteasome inhibitors (131). VLX1570 is a clinical-stage derivative of b-AP15 that similarly targets *USP14* and *UCHL5*, showing potent anti-myeloma activity in preclinical studies (132). This agent has entered early-phase clinical trials in patients with relapsed/refractory multiple myeloma (133), representing one of the earliest attempts to translate DUB inhibition into cancer therapy. However, its clinical development was halted due to dose-limiting toxicities. These toxicities primarily stem from its Michael acceptor-mediated off-target covalent binding and the narrow therapeutic window associated with pan-inhibition. To overcome these bottlenecks, drug development has shifted toward highly selective, non-covalent allosteric inhibitors.

Another extensively investigated compound is the selective USP7 inhibitor P5091. USP7 stabilizes multiple oncogenic substrates, including MDM2, β-catenin, EZH2, c-Myc-associated regulatory factors, and stemness-related transcriptional networks, thereby promoting CSC survival, facilitating EMT, participating in DNA damage repair, and mediating tumor drug resistance (134–137). Preclinical studies have demonstrated that P5091 induces apoptosis and inhibits tumor growth in hematological malignancies, including multiple myeloma, chronic lymphocytic leukemia, and lymphoma (138–140). Moreover, USP7 inhibition has been shown to attenuate stem-like phenotypes, impair homologous recombination repair, and sensitize tumor cells to DNA-damaging agents, providing a potential strategy for reversing chemotherapy and radiotherapy resistance. In parallel, next-generation USP7 inhibitors and related derivatives, such as FT671, FT827, GNE-6640, and GNE-6776, have further advanced the concept of selective targeting of DUBs (141). Other DUB inhibitors have also demonstrated therapeutic potential in experimental models. For example, mitoxantrone has been reported to inhibit USP11, thereby interfering with DNA damage repair signaling and sensitizing tumor cells to poly(ADP-ribose) polymerase (PARP) inhibitors (142). Compounds such as ML323 can inhibit USP1 and destabilize substrates involved in DNA repair and stemness maintenance, representing a potentially effective strategy to enhance the efficacy of platinum agents, PARP inhibitors, and radiotherapy (143). Similarly, inhibitors targeting USP10, USP13, and UCHL1 are currently in active preclinical research stages.

Therapeutic targeting of DUBs is closely linked to EMT, CSC biology, and metabolic plasticity. Beyond directly inducing tumor cell death, DUB-targeted strategies may effectively disrupt plastic cell states that drive metastasis and disease relapse. Numerous DUBs stabilize transcription factors that induce EMT and their upstream signaling pathways. For instance, USP22, USP29, USP4, and USP7 have all been implicated in maintaining EMT-like and stem-like phenotypes across various tumor types. Pharmacological inhibition of these DUBs can suppress tumor cell migration and invasion, reduce tumorsphere formation, and impair tumor-initiating capacity. MitoCur-1 is a curcumin derivative initially designed to selectively target tumor mitochondria and induce redox imbalance; it has recently been identified as a novel USP14 inhibitor. It exerts antitumor activity by inducing ferroptosis in melanoma cells and significantly sensitizes vemurafenib-resistant cells (144). Given the significant phenotypic plasticity of CSCs, DUB inhibition alone is typically insufficient to eradicate tumors. Therefore, these findings underscore the necessity of developing rational combination therapies to enhance anti-tumor efficacy. First, dual-targeting strategies can be employed within the DDR network. For instance, specific DUBs such as USP1 are critical for double-strand break repair. Combining USP1 inhibitors (such as KSQ-4279) with PARP inhibitors or platinum-based chemotherapy can induce synthetic lethality, effectively reversing chemoresistance in BRCA-proficient tumors. Second, therapeutic regimens can be designed to co-target the DUB-metabolism-ferroptosis axis. Since CSCs heavily rely on metabolic reprogramming and ferroptosis evasion to cope with therapeutic stress, combining DUB inhibitors with metabolic antagonists represents a highly promising strategy. For example, the recently discovered mechanism by which the USP24-DHODH axis drives ferroptosis resistance in TNBC provides a theoretical basis for combining selective USP24 inhibitors with ferroptosis inducers, a method that can metabolically deplete CSCs and dismantle their plasticity (Figure 4).

**Figure 4:**
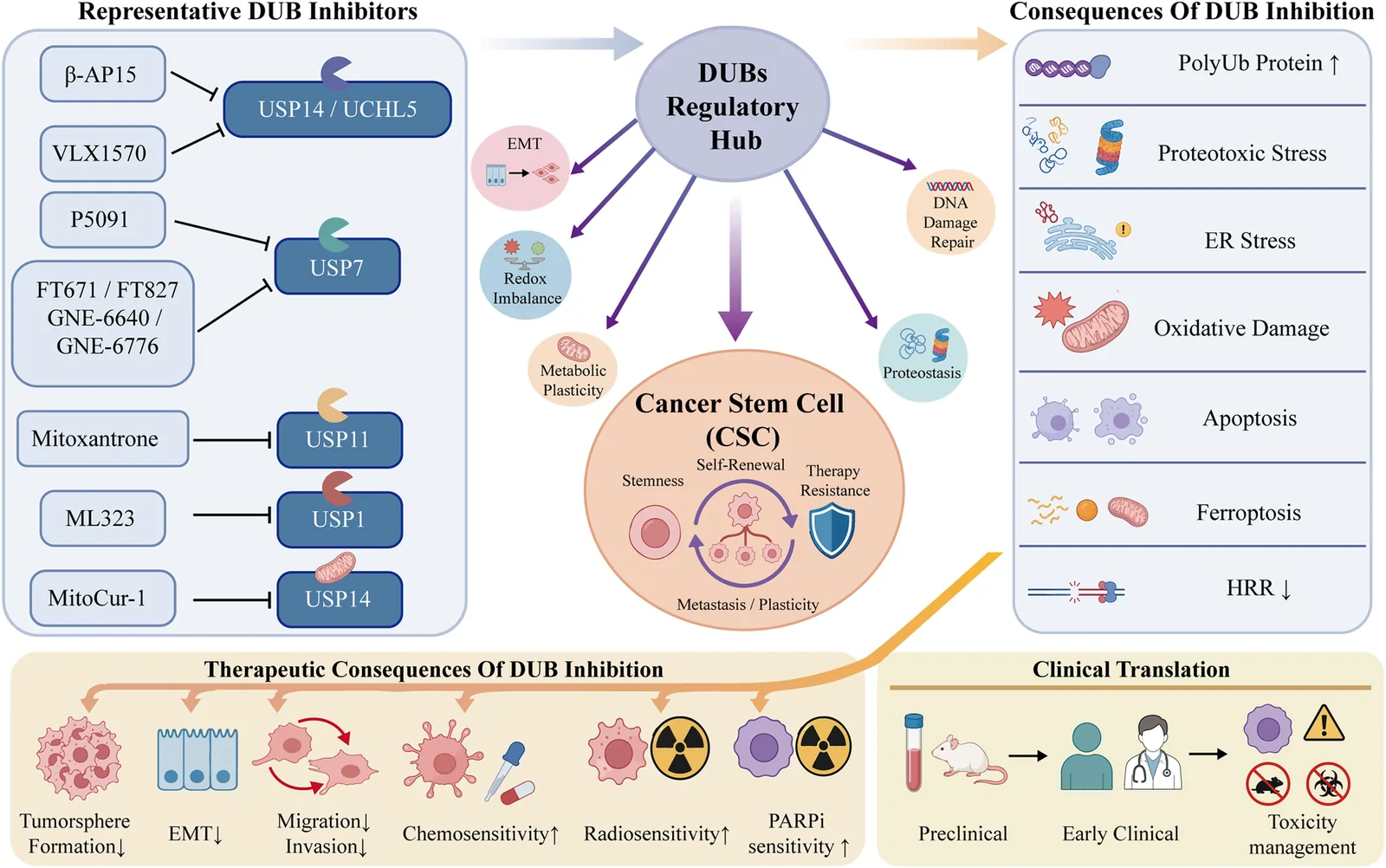
Therapeutic potential of targeting DUBs in CSCs. DUBs have emerged as attractive therapeutic targets because they occupy central nodal positions in the molecular networks that sustain CSC phenotypes, including EMT, metabolic plasticity, proteostasis, DNA damage repair, and resistance to therapy. By stabilizing key signaling mediators, transcription factors, and metabolic regulators, DUBs support CSC maintenance, invasion, metastatic fitness, and adaptive survival under therapeutic stress. Pharmacological inhibition of DUBs, such as USP14, UCHL5, USP7, USP11, and USP1, disrupts these regulatory circuits and can induce the accumulation of polyubiquitinated proteins, proteotoxic stress, oxidative damage, apoptosis, ferroptosis, defective DNA repair, and loss of stem-like properties. Representative DUB inhibitors include b-AP15, VLX1570, P5091, FT671, FT827, GNE-6640, GNE-6776, mitoxantrone, ML323, and MitoCur-1. Collectively, DUB-targeted strategies may suppress tumor progression, overcome therapeutic resistance, and improve the efficacy of combination treatments, although toxicity and clinical selectivity remain major translational challenges. PolyUb, polyubiquitinated; HRR, homologous recombination repair; upward arrows indicate increase, whereas downward arrows indicate reduction or impairment.

## 9 Conclusion and future prospects

The considerable plasticity of CSCs remains an important challenge to achieving durable therapeutic responses. In this context, DUBs may serve as regulatory nodes involved in CSC homeostasis. Rather than functioning solely as regulators of protein stability, DUBs may also modulate the context-dependent interplay between EMT and metabolic reprogramming. By stabilizing selected components of developmental signaling pathways (such as *Wnt*/β-catenin, *Hh, Notch*, and TGF-β) and chromatin modifiers, DUBs may connect the phenotypic changes associated with invasion to metabolic adaptations that support survival under stressful tumor microenvironmental conditions. Changes in DUB localization, activity, or substrate recognition in response to microenvironmental stress may therefore contribute to CSC plasticity and therapeutic resistance.

### 9.1 Central hypothesis and key open questions

Importantly, DUB-substrate interactions are not simple “one DUB, one substrate” relationships but are highly context-dependent, shaped by subcellular localization, scaffold proteins, and cellular metabolic states. This regulatory complexity creates several challenges for therapeutic translation. Broad-spectrum DUB inhibition may cause systemic toxicity, while structural homology within catalytic domains hinders the development of highly selective small-molecule inhibitors. Consequently, we propose that precise targeting of the DUB-EMT-metabolism axis—potentially using emerging approaches such as deubiquitinase-targeting chimeras (DUBTACs) or rational combination strategies—may provide a means of limiting CSC-associated plasticity. By shifting from global enzyme blockade to substrate-directed ubiquitin editing, DUBTACs can selectively restore proteins that antagonize EMT, inhibit stemness, or sensitize tumors to therapeutic stress. However, whether these approaches can sufficiently restrict CSC metabolic and phenotypic plasticity while avoiding off-target effects and functional redundancy remains to be established.

To overcome the remaining translational barriers, future research should complement descriptive substrate catalogs with mechanistic studies of DUB selectivity. Several key questions remain to be addressed:

1. Regarding dynamic substrate specificity, it is essential to determine how individual DUBs alter their substrate preferences (e.g., between EMT-transcription factors and metabolic enzymes) in response to distinct microenvironmental cues (such as hypoxia and acidic pH) or therapeutic stress.
2. For spatiotemporal mapping, future studies should determine whether integrating spatial transcriptomics with single-cell metabolic profiling can define the precise spatiotemporal trajectories of DUB activity within heterogeneous CSC subpopulations during metastasis and acquired resistance.
3. Concerning the translational optimization of DUBTACs, future studies should determine how to improve their tissue penetration, target selectivity, and bio-stability of these molecules to achieve potent in vivo efficacy against CSCs without triggering broad UPS toxicity.
4. Regarding patient-specific model validation, future studies should determine whether integrating patient-derived organoids (PDOs) with single-cell multi-omics approaches can adequately model the spatiotemporal patterns of DUB activity within patient-specific tumor microenvironments.

Ultimately, addressing these questions may support the identification of predictive biomarkers and more informative patient stratification, as well as the evaluation of rational combination strategies integrating targeted DUB modulation with conventional chemoradiotherapy, targeted agents, or immunotherapy.

## Glossary

ALDH1A3: aldehyde dehydrogenase 1A3
BCC: basal cell carcinoma
BCSCs: breast cancer stem cells
CDH1: E-cadherin
CDH2: N-cadherin
Ci: cubitus interruptus
CNS: central nervous system
CSCs: cancer stem cells
DDR: DNA damage response
DUBs: deubiquitinating enzymes
EMT: epithelial-mesenchymal transition
EMT-TFs: EMT transcription factors
FAD: flavin adenine dinucleotide
FoxA2: forkhead box protein A2
Fzd: Frizzled
GBM: glioblastoma
GFAP: glial fibrillary acidic protein
Gli: glioma-associated oncogene homolog
GSCs: glioblastoma stem cells
HEK293T: human embryonic kidney 293T
Hh: Hedgehog
HIF-1α: hypoxia-inducible factor-1α
HNF4: hepatocyte nuclear factor 4
HOXA10: homeobox A10
ID1: inhibitor of differentiation 1
IL-8: interleukin-8
JAMMs: JAB1/MPN/Mov34 metallo-proteases
LRP6: low-density lipoprotein receptor-related protein 6
LSD1: lysine-specific demethylase 1
MEFs: mouse embryonic fibroblasts
MJDs: Machado-Joseph disease proteases
NB: neuroblastoma
NICD: Notch intracellular domain
OTUs: ovarian tumor proteases
p-CHEK1: phosphorylated checkpoint kinase 1
PDAC: pancreatic ductal adenocarcinoma
PFS: progression-free survival
PKA: protein kinase A
PTCH: patched
PYGL: glycogen phosphorylase L
RNAi: RNA interference
ROS: reactive oxygen species
SHH: Sonic hedgehog
SMO: Smoothened
Sufu: Suppressor of fused
T-ALL: T-cell acute lymphoblastic leukemia
TRRAP: transformation/transcription domain-associated protein
UCHs: ubiquitin C-terminal hydrolases
UPS: ubiquitin-proteasome system
USPs: ubiquitin-specific proteases
VIM: vimentin
β-TrCP: β-transducin repeat-containing protein.
